# ACPNet: A Deep Learning Network to Identify Anticancer Peptides by Hybrid Sequence Information

**DOI:** 10.3390/molecules27051544

**Published:** 2022-02-24

**Authors:** Mingwei Sun, Sen Yang, Xuemei Hu, You Zhou

**Affiliations:** 1 Key Laboratory of Symbol Computation and Knowledge Engineering of Ministry of Education, College of Computer Science and Technology, Jilin University, Changchun 130012, China; sunmw19@mails.jlu.edu.cn (M.S.); huxm18@mails.jlu.edu.cn (X.H.); 2 College of Computer Science and Technology, Tonghua Normal University, Tonghua 134000, China; 3 School of Computer Science and Artificial Intelligence Aliyun School of Big Data School of Software, Changzhou University, Changzhou 213164, China; ys@cczu.edu.cn; 4 College of Software, Jilin University, Changchun 130012, China

**Keywords:** anticancer peptides, multi-view information, deep learning

## Abstract

Cancer is one of the most dangerous threats to human health. One of the issues is drug resistance action, which leads to side effects after drug treatment. Numerous therapies have endeavored to relieve the drug resistance action. Recently, anticancer peptides could be a novel and promising anticancer candidate, which can inhibit tumor cell proliferation, migration, and suppress the formation of tumor blood vessels, with fewer side effects. However, it is costly, laborious and time consuming to identify anticancer peptides by biological experiments with a high throughput. Therefore, accurately identifying anti-cancer peptides becomes a key and indispensable step for anticancer peptides therapy. Although some existing computer methods have been developed to predict anticancer peptides, the accuracy still needs to be improved. Thus, in this study, we propose a deep learning-based model, called ACPNet, to distinguish anticancer peptides from non-anticancer peptides (non-ACPs). ACPNet employs three different types of peptide sequence information, peptide physicochemical properties and auto-encoding features linking the training process. ACPNet is a hybrid deep learning network, which fuses fully connected networks and recurrent neural networks. The comparison with other existing methods on ACPs82 datasets shows that ACPNet not only achieves the improvement of 1.2% Accuracy, 2.0% F1-score, and 7.2% Recall, but also gets balanced performance on the Matthews correlation coefficient. Meanwhile, ACPNet is verified on an independent dataset, with 20 proven anticancer peptides, and only one anticancer peptide is predicted as non-ACPs. The comparison and independent validation experiment indicate that ACPNet can accurately distinguish anticancer peptides from non-ACPs.

## 1. Introduction

Currently, cancer is an enormous threat to human health, reported by the International Agency for Research on Cancer (IARC) [1], leading to rapidly increasing mortality every year, reaching 18.1 million new cases and 9.6 million cancer deaths in 2018 alone. Moreover, the type of common cancer is increasing, including lung, breast, prostate, colorectal and so on. However, the diagnosis and therapy of cancer are challenging, which hinders the therapy process, such as surgery, radiotherapy, chemotherapy, and targeted therapy. Furthermore, traditional methods for the treatment of cancer always meet costly and high-risk problems, accompanied by the risk of tissue death, drug resistance and other serious side effects [2]. Fortunately, increasing evidence shows that some novel anticancer agents, for some peptides for instance, become novel and safe for therapy [3]. For example, peptide p28 is a post-translational, multi-target anticancer agent that preferentially enters a wide variety of solid tumor cells and binds to both wild-type and mutant p53 protein, inhibiting constitutional morphogenic protein 1 (Cop1)-mediated ubiquitination and proteasomal degradation of p53, which results in increased levels of p53 and induces cell-cycle arrest at G2/M and eventual apoptosis, which results in tumor cell shrinkage and death [4]. LL-37, a novel anticancer peptide, has a net positive charge and is amphiphilic, and can eliminate pathogenic microbes directly, via electrostatic attraction towards negatively charged bacterial membranes. Several studies have shown that LL-37 participates in various host immune systems, such as inflammatory responses and tissue repair [5]. Peptide-based therapeutics are more affordable, tolerable and safe, which are considered to be advanced therapy strategies [6]. Therefore, it is a significant step to rapidly and effectively find useful anticancer peptides (ACPs). Although there are many experimental methods to identify ACPs, they are usually laborious, expensive, time consuming and hard to achieve in a high-throughput manner. Furthermore, with the rapid development of data science, it is very desirable to design machine learning-based methods to identify ACPs [7].

Over the past few years, a dozen computational methods have been proposed to identify ACPs, including k-nearest neighbor (KNN), support vector machine (SVMs), random forest (RF) and so on. In 2019, Boopathi, V. et al. proposed a machine learning model to predict ACPs, called mACPpred [7], which uses seven types of encoding features, including amino acid composition (AAC), dipeptide composition (DPC), composition-transition-distribution (CTD), quasi-sequence-order (QSO), amino acid index (AAIF), binary profile (NC5) and conjoint triad (CTF) to represent a peptide sequence and cooperate with an SVM model to predict ACPs. In 2020, Li Qingwen, et al. employed five types of peptide sequence features, including amino acid composition (AAC), conjoint triad (CT), pseudo-amino acid composition (PAAC), grouped amino acid composition (GAAC) and C/T/D, and then fused multiple machine learning methods, containing SVM, RF, and LibD3C, to identify ACPs [8]. In 2020, Ge Ruiquan et al. proposed a machine model called EnACP, which introduces sequence composition, sequence-order, physicochemical properties, etc. to encode a peptide sequence and input the important feature selected by multiple ensemble classifiers to an SVM model to predict ACPs [9]. These methods try to find effective and useful features to represent a peptide and combine a high-performance machine model to identify ACPs. Another way to identify peptides is by introducing a deep learning model to identify ACPs from the raw sequence of peptides. In 2019,Yi Haichent et al. proposed a deep learning long short-term memory (LSTM) neural network model, called ACP-DL [10], which developed an efficient feature representation approach by integrating binary profile features, k-mer sparse matrix of the reduced amino acid alphabet and then implemented a deep LSTM model to identify ACPs. In 2021, Chen Xiangan et al. proposed an ACP prediction model, called ACP-DA [11], which uses data augmentation for insufficient samples and trains a multilayer perception model to improve the prediction performance. In 2020, Yu Lezheng et al. found that the recurrent neural network with bidirectional long short-term memory cells is a superior architecture to identify ACPs and implement a sequence-based deep learning tool, called DeepACP [12], to accurately predict ACPs.

Although these methods can predict ACPs accurately, the accuracy performance of existing methods still needs to be improved. It is also a challenge to represent peptide sequences to numerical vectors and further improve the prediction accuracy of ACPs. Therefore, in this paper, we propose a hybrid deep learning-based model, called ACPNet, which employs the raw peptide sequence and carefully selected sequence features as input, to fit recurrent neural networks, and fully connected network to further improve the predicting performance.

## 2. Materials and Methods

### 2.1. Materials

The sequences of ACP and non-ACP are downloaded from this research used in [12]. Three datasets are introduced including ACPs250, ACPs82, ACPs20. ACPs250 contains 250 ACPs and 250 non-ACPs sequence samples, ACPs82 is made up of 82 ACPs and 82 non-ACPs sequence samples, ACPs20 contains 10 ACP samples and 10 non-ACP samples. ACPs250 dataset is split into training dataset and validation dataset with 80% and 20% respectively. To further validate the performance of ACPNet, we conducted experiments on an independent test dataset ACPs82. Furthermore, ACPs20, another independent dataset, is introduced to further prove the performance of ACPNet. These datasets are listed in Table 1.

### 2.2. Methods

#### 2.2.1. Features Construction

To further improve the prediction accuracy of ACPs, in this work, we employed three hybrid kinds of features to encode a peptide sequence to a numerical vector, including peptide sequence features, peptide physicochemical properties and automatic embedding features, which are listed in Table 2.

##### Sequence Features

The concept of PAAC (pseudo amino acid composition) [13] was introduced to avoid completely losing the sequence-order information [1]. In contrast with the conventional amino acid composition (AAC) that contains 20 components, the PAAC contains a set of greater than 20 discrete factors, where the first 20 represent the components of its conventional amino acid composition while the additional factors incorporate some sequence-order information via various pseudo components.

PAAC can be represented by ${P=[p}_{1}{,\;p}_{2},\;\ldots {,\;p}_{20}{,\;p}_{20+1},\;\ldots {,\;p}_{20+\lambda }{]}^{T},\;(\lambda \;<\;L)$, where λ is an integrated parameter set by a user with recommended value 10, $p_{u}$ is calculated by Equation (1)

(1) $$p_{u}=\{\begin{array}{c} \frac{f_{u}}{\sum_{i=1}^{20}f_{i}+w\sum_{k=1}^{\lambda }\tau_{k}},\;(i\leq u\leq 20)\\ \;\;\;\;\;\;\;\;\;\;\;\;\;\;\;\frac{{w\tau }_{u-20}}{\sum_{i=1}^{20}f_{i}+w\sum_{k=1}^{\lambda }\tau_{k}},\;(21\leq u\leq 20+\lambda ) \end{array}$$

where $f_{i}$ is the frequency of each amino acid in a protein, w is the weight factor set by a user with default value 0.05,$\tau_{k}$ is the k-th tier correlation factor that reflects the sequence order correlation between all the k-th most contiguous residues as formulated by Equation (2).

(2) $$\tau_{k}=\frac{1}{L-k}\sum_{i=1}^{L-k}J_{i,i+k}\;\;\;\;\;\;\;\left(k\text{<}L\right)$$

where $J_{i,i+k}$ can be calculated by Equation (3).

(3) $$J_{i,i+k}=\frac{1}{\Gamma }\sum_{q=1}^{\Gamma }{{[\Phi }_{q}{(R}_{i+k}{)-\Phi }_{q}{(R}_{i})]}^{2}$$

where $\Phi_{q}{(R}_{i})$ is the q-th function of the amino acid $R_{i}$ and Γ the total number of functions considered, such as hydrophobicity value, hydrophilicity value, and side-chain mass of amino acid.

Another two features are peptide sequence length and Shannon entropy of peptide sequences [14]. The Shannon entropy of peptide sequences can be obtained by Equation (4).

(4) $$\mathrm{Shannon}\;\mathrm{entropy}(\mathrm{seq})=-\sum_{i=1}^{20}f_{i}\log(f_{i})$$

##### Peptide Physicochemical Properties

Peptide, a short chain of amino acids, exhibits many similar properties to proteins and the physicochemical properties have close relations with protein functions. Therefore, three physicochemical properties of peptides are introduced to represent peptide sequences including Gravy [15], Molecular_weight [16] and Charge_at_pH [17]. Gravy feature is used to describe peptide gravy according to Kyte and Doolittle, Molecular_weight is employed to represent peptide molecular weight, Charge_at_pH is introduced to calculate the charge number of a protein when given pH set 10.

##### Embedding Features

**Figure 1:**
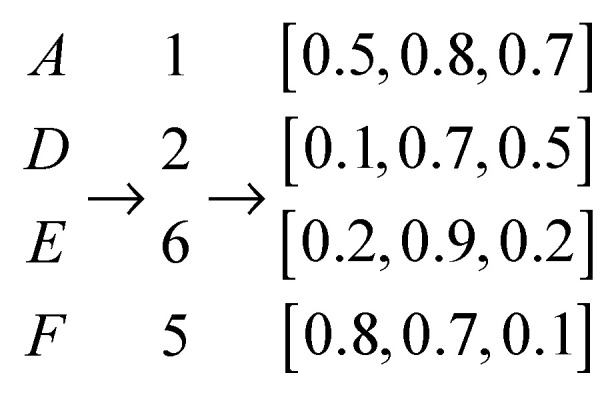
The process to auto-embed a peptide sequence to a matrix.

A peptide can be seen as a sentence containing ‘word’ as ‘A’, ‘C’, ‘D’, ‘E’, ‘F’, ‘G’, ‘H’, ‘I’, ‘K’, ‘L’, ‘M’, ‘N’, ‘P’, ‘Q’, ‘R’, ‘S’, ‘T’, ‘V’, ‘W’, ‘Y’, which represent 20 kinds of amino acids. To encode a peptide sequence to a numerical vector, a peptide is converted to a numerical vector by amino acid index with the same length of peptide length such as [1,2,4,…,20,5]. The corresponding map is ‘A’→1, ‘C’→2, …, ‘Y’→20. After a peptide is converted to a numerical vector, the following step is to map each index to a user-defined dimension vector. The embedding process is to turn positive integers (indexes) into dense vectors. For a peptide sequence “ADGF” is an example with a user-defined dimension as three, the representation process is shown in Figure 1.

#### 2.2.2. Model Structure

##### Overall Workflow

**Figure 2:**
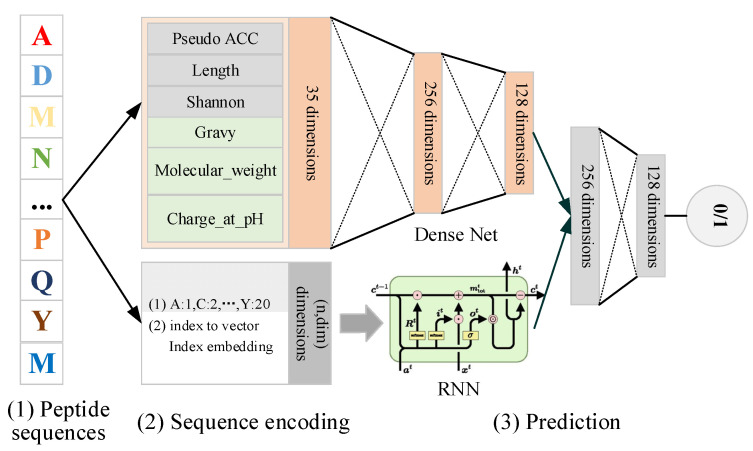
The overall workflow of ACPNet.

Deep learning technology has obtained numerous achievements in many bioinformatics applications [18,19,20]. Therefore, in this paper, we propose a hybrid deep learning-based model, named ACPNet, for predicting ACPs. The overall workflow is shown in Figure 2. The detailed structure of ACPNet is in Supplementary Figure S1.

The manually selected features and auto-embedding features are fed to a fully connected neural network (Dense Network) and recurrent neural network (RNN) [21] respectively, and then the result of the previous process is merged for the final prediction. Overall, ACPNet combines Dense Network and RNN to construct a hybrid deep learning-based model to identify ACPs, which not only consider the importance of manually selected features but also automatically learn the potential features from raw peptide sequences as well.

##### Prediction Model Constructed by RNN and Dense Networks

RNN and Dense network, as the two most significant deep learning models, are widely applied on time serial and feature independent issues respectively. LSTM [22], a kind of implementation of RNN, is usually employed to further automatically extract potential features from the time serial vectors. The kernel process of LSTM can be illustrated by Equation (5).

(5) $$\begin{array}{l} f_{t}{=\sigma }_{g}{(W}_{f}x_{t}{+U}_{f}h_{t-1}{+b}_{f})\\ i_{t}{=\sigma }_{g}{(W}_{i}x_{t}{+U}_{i}h_{t-1}{+b}_{i})\\ {ο}_{t}{=\sigma }_{g}{(W}_{ο}x_{t}{+U}_{ο}h_{t-1}{+b}_{ο})\\ c_{t}{=f}_{t}\circ c_{t-1}{+i}_{t}\circ \sigma_{c}{(W}_{c}x_{t}{+U}_{c}h_{t-1}{+b}_{c})\\ h_{t}{=ο}_{t}\circ \sigma_{h}{(c}_{t}) \end{array}$$

where, $x_{t}$ is the input vector, $h_{t}$ is output vector, $c_{t}$ is the cell state vector, W, U and b are the learning parameters, $f_{t}$ is a forget gate vector to remember old information, $i_{t}$ is input gate vector to acquire new information, ${ο}_{t}$ is output gate vector as output candidate, $\sigma_{g}$, $\sigma_{c}$ and $\sigma_{h}$ are three activate functions. LSTM is employed in ACPNet for the belief that the peptide sequence can be seen as time series data. The dense network processes 1-dimension data with independent features expertly, therefore a Dense network is employed to process manual-selected features and the final prediction.

##### Implementation of ACPNet

ACPNet is implemented by Tensorflow 2.5.0 and all scripts are written by Python 3.8. ACPNet is running on a personal computer with 4.3 GHz, 8 core CPU, and 64 GB RAM under an open Linux operating system.

#### 2.2.3. Performance Evaluation of ACPNet

ACPNet is evaluated by the widely used standard performance metrics, which are Accuracy, F1-score, Recall, Precision and Matthews correlation coefficient (MCC). These evaluation metrics are defined as follows:

$$\mathrm{Accuracy}=\frac{\mathrm{TP}+\mathrm{TN}}{\mathrm{TP}+\mathrm{TN}+\mathrm{FP}+\mathrm{FN}}\;F1-\mathrm{score}=\frac{2\mathrm{TP}}{2\mathrm{TP}+\mathrm{FP}+\mathrm{FN}}\;\mathrm{Recall}=\frac{\mathrm{TP}}{\mathrm{TP}+\mathrm{FN}}\;\mathrm{Precision}=\frac{\mathrm{TP}}{\mathrm{TP}+\mathrm{FP}}\;\begin{array}{l} \mathrm{Matthews}\;\mathrm{Correlation}\;\mathrm{coefficient}\;\left(\mathrm{MCC}\right)\\ =\frac{\mathrm{TP}*\mathrm{TN}-\mathrm{FP}*\mathrm{FN}}{\sqrt{\left(\mathrm{TP}+\mathrm{FN})*(\mathrm{TP}+\mathrm{FP})*(\mathrm{TN}+\mathrm{FP})*(\mathrm{TN}+\mathrm{FN}\right)}} \end{array}$$

where TP, FP, TN and FN represent the true positives, false positives, true negatives and false negatives, respectively. We also plot the receiver operating characteristic curves (ROC) and computed Area Under the Curve (AUC) to show the performance of ACPNet.

## 3. Results

### 3.1. The Effects of Feature Combination

To explore the effect of the combination of manually selected features and automatic learning features, the performances conducted by three types of combination are compared on ACPs250 (as training dataset), and ACPs82 (as test dataset). The compared results are listed in Table 3, and we found that the hybrid-fused features model shows better performance on multiple metrics. Note, in terms of MCC, the feature-fused model surpasses the manually selected feature model by more than 16%, and on other metrics, the feature-fused model shows advanced performance as well. The results indicate that the combination of two types of features play a positive and reinforced role in distinguishing between ACPs and non-ACPs.

### 3.2. Manually Selected Features Importance Rank

**Figure 3:**
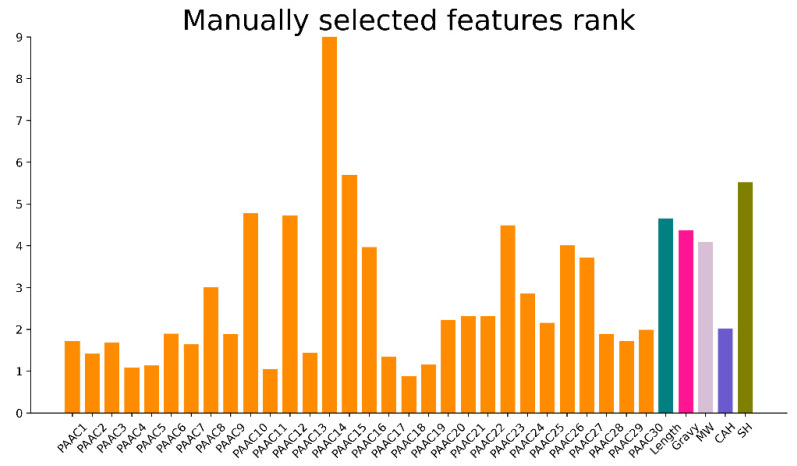
The importance rank of manually selected features.

To further show the importance of each manually selected feature, CatBoost [23], an ensemble machine learning framework, was introduced to calculate the importance score of each feature. Figure 3 shows the importance score of each manually selected feature. Length, Gravy, MW (Molecular_weight), and SH (Shannon entropy) obtained a relatively high score and surpassed the majority of PAAC’s features. The PAAC features also show a positive contribution. The PAAC features get the best score at the 14th feature and show not particularly important contributions at the 18th feature. Besides, each PAAC feature provides different contributions to identify ACP. Overall, the manually selected features contribute to the classification of ACPs and non-ACPs.

### 3.3. Feature Visualization

**Figure 4:**
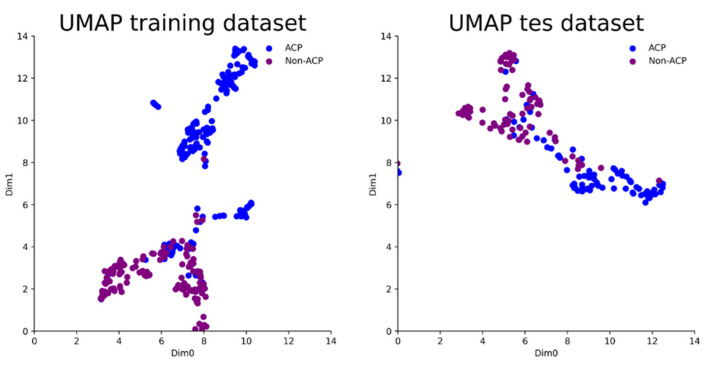
The importance rank of manually selected features.

We use Uniform Manifold Approximation and Projection (UMAP) [24] to visualize the distribution of ACPs and non-ACPs by media vector, generated on the ACPNet inner layer into two-dimensional space. Figure 4 illustrates that ACPs and non-ACPs in the training and test dataset can be easily classified by these features, which reconfirms that the constructed features contribute to the identification of ACPs and non-ACPs.

### 3.4. Performance Comparison of Models on Independent Datasets

To show the advance of ACPNet, traditional machine learning- and deep learning-based methods are employed as comparisons. For a fair comparison, the same training dataset, ACP250s, and independent test dataset, ACP82s, were used to train and test all methods. For traditional machine learning, SVM, RF, and CatBoost are introduced for the comparisons. Note, the auto embedding features are replaced by the index encoding for peptide sequence because the auto embedding links the training process. Therefore, the index encoding method and manually selected features are employed to encode a peptide for traditional machine learning. The comparison results of traditional machine learning are listed in Table 4.

For the comparison with the deep learning-based model, seven existing models are employed as the comparisons, which include AntiCP [25], Hajisharifi [26], iACP [27] and ACPred-FL [28], CNN (Convolutional Neural Network) [29], CNN+RNN, DeepACP [12]. AntiCP contains two models, which are AntiCP_ACC and Anticp_DPC, both based on SVM. AntiCP_ACC is built by amino acid composition features, while Anticp_DPC is constructed by dipeptide composition features. Hajisharifi combines two integrative SVM-based classification models for the prediction of anticancer peptides on the base of local alignment kernel and PAAC parameters. Further, iACP employs g-gap dipeptide components and SVM to predict ACPs. CNN is fed one-hot encoding matrixes to identify ACPs. For the CNN+RNN model, the input is the same as the CNN model, but after the CNN part, an RNN model links the following process. DeepACP, an end-to-end method, tries to fuse the peptide sequence encoding and training process to identify the ACPs. In the same way as the comparison with traditional machine learning, the ACP250s dataset and independent test dataset, ACP82s, are used to train and test all methods. The comparison results of deep learning-based methods are listed in Table 5. From Table 5, we can find that ACPNet shows better performance compared with the other seven methods in multiple metrics. In terms of precision, ACPNet is slightly lower than ACPred-FL, but shows nearly 7% improvement in Recall and achieves a more balanced performance. For other methods, except ACPred-FL, ACPNet obtained more than 6%, 6%, 10%, 3.5%, and 12% improvement, in terms of accuracy, F1-score, recall, precision and MCC. Overall, ACPNet shows higher performance compared with existing methods.

**Figure 5:**
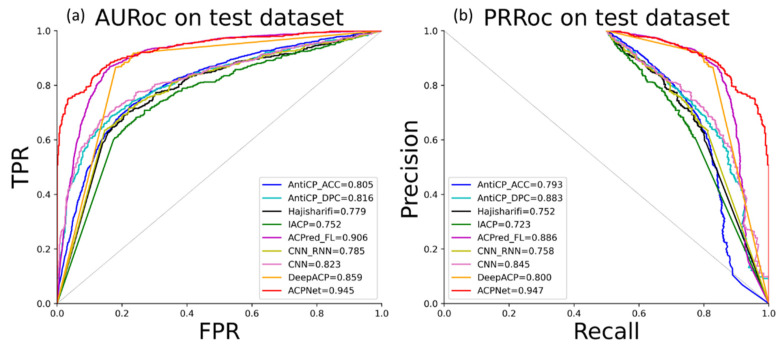
AURoc (**a**) and PRRoc (**b**) on test dataset.

Furthermore, we also plot the receiver operating characteristic curves (ROC) to further show the performance of ACPNet, shown in Figure 5, with AUC at 0.945 and PRAUC (Area Under the Precision–Recall Curve) [30] at 0.947, respectively. Figure 5 indicates that ACPNet shows better performance both on AUC and PRAUC.

### 3.5. Independent Validation

Furthermore, ACPNet is verified on an independent dataset, with 10 ACPs and 10 non-ACPs, and only one proven ACP is predicted as non-ACP, while all non-ACPs are predicted as non-ACPs. The independent validation results are listed in Table 6. Most ACPs obtained a higher score, large than 0.7. If the predicting score is larger than 0.5, the corresponding peptide will be treated as an ACP. The independent validation results indicate that ACPNet can accurately predict ACPs.

## 4. Discussion

Cancer is one of the most dangerous threats to human health. Anticancer peptides could be novel agents for the therapy of cancers [33]. Therefore, accurately identifying anticancer peptides is a key step for the therapy. Nowadays, although deep neural network models have been developed to predict ACP, the accuracy still needs to improve. Thus, in this study, we proposed a hybrid deep learning-based model, called ACPNet, to distinguish ACPs from non-ACPs. For the feature construction, three types of features were introduced. The first type of features are manually selected features, which include PAAC, Length, and Shannon entropy, calculated from peptide sequence information. The second type of features are Molecular_weight, Charge_at_pH, Gravy, which source from peptide physicochemical properties. The third type of features are autoencoding features, which link the training process by encoding each amino acid index to a vector. In part 3.3, the performance of the combination of three types of features is compared. The comparison results show that the combination of three types of features play a positive role in identifying ACPs. Each manually selected feature, including sequence information and peptide physicochemical properties features, is relatively independent, so a fully connected neural network is employed to train the manually selected features. Autoencoding features of peptides can be seen as time serial data, which fit the learning pattern of BiLSTM. Therefore, Sequence information and peptide physicochemical properties are merged to feed fully connected networks. Autoencoding features are input into a BiLSTM network [34]. After passing the two types of networks, the media vectors are merged to feed a fully connected network for the final prediction. ACPNet is a hybrid deep learning network, which fuses the advance of two types of networks to build the neural network structure and full use of three kinds of information features as inputs, to improve the prediction accuracy of ACPs. For a comparison with other existing methods, ACPNet not only shows higher performance in multiple metrics, including Accuracy, F1-score, Recall, Precision and MCC, but also shows balanced performance metrics. This means that ACPNet may show better robustness for future identification. Furthermore, media vectors generated on the ACPNet inner layer are compressed into two dimensions, to further show the entire performance directly. The visualization results indicate that three different types of features of peptides and hybrid deep learning-based models can accurately distinguish ACPs from non-ACPs. Furthermore, ACPNet is verified on an independent dataset, with 10 ACPs and 10 non-ACPs. Only one proven ACP was predicted as a non-ACP, and all the non-ACPs were predicted as non-ACPs. The independent validation results indicate that ACPNet can accurately distinguish ACPs from non-ACPs. The learning pattern of ACPNet also fits other peptide-related works, which may provide a useful clue in solving these problems. ACPNet also meets some limitations. For example, ACPNet does not provide a user-friendly web server, which may make it difficult for some people who don’t know how to program. Furthermore, ACPNet does not consider the different types of cancer, which may generate bias. In the future, we will try to build a new ACP prediction model, based on the different types of cancer, and provide a user-friendly web server to the public.

## 5. Conclusions

In this work, we proposed a deep learning-based method, ACPNet, to identify ACPs, by combining a hybrid deep learning-based model with manually selected features and automatic encoding features as the input. For the feature construction, three types of features were introduced, namely, peptide sequence component information features, peptide physicochemical properties and auto-encoding features. Three different types of features play a positive role in distinguishing ACPs and non-ACPs. A fully connected network and recurrent neural network were introduced to process the constructed features. Compared with existing methods, ACPNet shows better and more balanced performance, with 1.2% accuracy, 2.0% F1-score, and 7.2% Recall improvement. On a three-part dataset, with 10 ACPs and 10 non-ACPs, ACPNet accurately predicts nine in ten ACPs. The series experiments show that ACPNet can accurately distinguish ACPs from non-ACPs.

## Figures and Tables

**Table 1:** Datasets for train, test and independent validation of ACPNet.

	ACPs Number	Non-ACPs Number	Average Length	Max Length	Min Length
ACPs250	250	250	27	97	11
ACPs82	82	82	27	207	11
ACPs20	10	10	24	47	13

**Table 2:** Three kinds of hybrid features to encode peptide sequences.

Feature Types	Feature Name	Dimensions
Sequence features	PAAC	30
	Length	1
	Shannon entropy	1
Peptide physicochemical properties	Gravy	1
	Molecular_weight	1
	Charge_at_pH(10)	1
Embedding features	Position embedding	50

**Table 3:** The performance results compared by three types of combinations on ACPs82.

	TP	TN	FP	FN	Accuracy	F1-Score	Recall	Precise	MCC	AUC	PRAUC
MS	65	68	14	17	81.0	80.7	79.2	82.2	0.622	0.841	0.832
AE	67	73	9	15	85.3	84.8	81.7	88.1	0.709	0.867	0.878
MS + AE	72	75	7	10	89.6	89.4	87.8	90.1	0.793	0.945	0.947

MS means manually selected features, AE means automatic learning features.

**Table 4:** Comparing results with traditional machine learning.

	TP	TN	FP	FN	Accuracy	F1-Score	Recall	Precision	MCC	AUC	PRAUC
SVM	60	63	22	19	75.0	75.5	73.1	75.9	0.500	0.775	0.763
RF	81	28	1	54	66.4	74.6	98.7	60.0	0.431	0.704	0.697
CatBoost	64	77	18	5	85.9	84.7	78.0	92.7	0.728	0.883	0.891
ACPNet	72	75	7	10	89.6	89.4	87.8	91.1	0.793	0.945	0.947

**Table 5:** Performance comparisons of ACPNet with the existing methods.

	TP	TN	FP	FN	Accuracy	F1-Score	Recall	Precision	MCC	AUC	PRAUC
AntiCP_ACC	56	71	26	11	77.4	75.2	68.3	83.6	0.558	0.805	0.793
Anticp_DPC	61	69	21	13	79.3	78.2	74.4	82.4	0.588	0.816	0.883
Hajisharifi	55	71	27	11	76.8	74.3	67.1	83.3	0.547	0.779	0.752
IACP	56	66	26	16	74.4	72.7	68.3	78.8	0.491	0.752	0.723
ACPred-FL	66	79	16	3	88.4	87.4	80.5	95.7	0.778	0.906	0.886
CNN-RNN	59	67	23	15	76.8	75.6	72.0	79.7	0.539	0.785	0.758
CNN	64	65	18	17	78.6	78.5	78.0	79.0	0.573	0.823	0.845
DeepACP	64	72	18	10	82.9	82.0	78.0	86.5	0.662	0.859	0.800
ACPNet	72	75	7	10	89.6	89.4	87.8	91.13	0.793	0.945	0.947

**Table 6:** The independent validation results of ACPNet.

Id	Sequence	Score	Label
1	KLWKKIEKLIKKLLTSIR	0.9999	ACP
2	YIWARAERVWLWWGKFLSL	0.9994	ACP
3	DLFKQLQRLFLGILYCLYKIW	0.8732	ACP
4	AIKKFGPLAKIVAKV	0.7043	ACP
5	RWNGRIIKGFYNLVKIWKDLKG	0.9620	ACP
6	KVWKIKKNIRRLLHGIKRGWKG	0.9993	ACP
7	GFWARIGKVFAAVKNL	0.9988	ACP
8	AFLYRLTRQIRPWWRWLYKW	0.4979	Non-ACP
9	RIWGKHSRYIKIVKRLIQ	0.9993	ACP
10	QIWHKIRKLWQIIKDGF	0.9997	ACP
11	CGESCVWIPCVTSIFNCKCKENKVCYHDKIP	0.0001	Non-ACP
12	SDEKASPDKHHRFSLSRYAKLANRLANPKLLETFLSKWIGDRGNRSV	0.2383	Non-ACP
13	DVKGMKKAIKGILDCVIEKGYDKLAAKLKKVIQQLWE	0.4986	Non-ACP
14	AGWGSIFKHIFKAGKFIHGAIQAHND	0.011	Non-ACP
15	ATCDLASGFGVGSSLCAAHCIARRYRGGYCNSKAVCVCRN	0.0032	Non-ACP
16	GWKIGKKLEHHGQNIRDGLISAGPAVFAVGQAATIYAAAK	0.0015	Non-ACP
17	FLGALIKGAIHGGRFIHGMIQNHH	0.4750	Non-ACP
18	FLPAIAGILSQLF	0.1818	Non-ACP
19	ALWMTLLKKVLKAAAKALNAVLVGANA	0.0052	Non-ACP
20	EGGGPQWAVGHFM	0.1243	Non-ACP

Note: the independent validation peptide sequences (1–10, ACP) and (11–20, non-ACP) source from [31,32] respectively.

## Data Availability

Publicly available datasets were analyzed in this study. Codes and data are available here: https://github.com/abcair/ACPNet accessed on 2 January 2022.

## Supplementary Materials

The following supporting information can be downloaded online. Figure S1: detailed structure of ACPNet.
