# Supplementary figures and images for: ACPNet: A Deep Learning Network to Identify Anticancer Peptides by Hybrid Sequence Information
Source: Molecules. 2022 Feb 24;27(5):1544. doi: 10.3390/molecules27051544 (PMC8912097; doi:10.3390/molecules27051544)

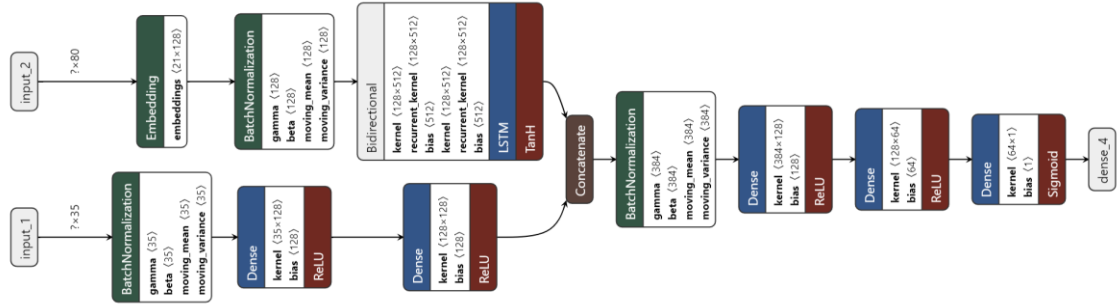

**Figure S1.** Detailed structure of ACPNet

Supplement: Supplementary file 1 [file molecules-27-01544-s001.zip › molecules-1563154-supplementary.pdf]
